# STRENGTHENING LATE-LIFE DEPRESSION COLLABORATIVE CARE THROUGH COMMUNITY ENGAGEMENT: CARE PARTNERS INITIATIVE

**DOI:** 10.1093/geroni/igz038.2120

**Published:** 2019-11-08

**Authors:** Ladson Hinton, Theresa J Hoeft, Stuart Henderson, Melissa M Gosdin, Laura Rath, Jurgen Unutzer

**Affiliations:** 1 University of California Davis, Sacramento, California, United States; 2 University of Washington, Seattle, Washington, United States; 3 Archstone Foundation, Long Beach, California, United States

## Abstract

Despite the availability of effective treatments for late life depression, many older adults with depression either do not access or fully engage in treatment. The goal of this study was to examine the feasibility and two-year outcomes from an Archstone Foundation funded Care Partners Initiative to strengthen depression care for adults 65 years of age and older. Seven sites throughout California implemented evidence-based collaborative care through partnerships between primary care organizations, community-based organizations (CBOs), and families of older adults with depression. Evaluation used a mixed-methods approach incorporating both qualitative and quantitative data. Of the seven sites, six formed partnerships between primary care clinics and CBOs and one site only focused on engaging family members in treatment. In the first two years, 274 patients were enrolled and rates of depression improvement were comparable to prior depression care effectiveness trials. Overall, 49% of patients at CBO sites interacted 3+ times with CBO staff/clinicians, while at the family site, 79% of patients had 3+ contacts including a family member. Using data from key informant interviews, focus groups, and site progress documents, seven core components were identified that facilitated successful implementation and delivery of partnered collaborative care, including three foundational components: strong stakeholder buy-in, effective patient engagement, and the promotion of depression treatment as a core value across organizations. Multiple complexities of partnering between primary care clinics and CBOs or families were identified. Challenges and lessons learned from this initiative will also be discussed.

